# Interspecific differences in how habitat degradation affects escape response

**DOI:** 10.1038/s41598-017-00521-0

**Published:** 2017-03-27

**Authors:** Mark I. McCormick, Bridie J. M. Allan

**Affiliations:** ARC Centre of Excellence for Coral Reef Studies and Department of Marine Biology and Aquaculture, James Cook University, Townsville, QLD 4811 Australia

## Abstract

Degradation of habitats is widespread and a leading cause of extinctions. Our study determined whether the change in the chemical landscape associated with coral degradation affected the way three fish species use olfactory information to optimize their fast-start escape response. Water from degraded coral habitats affected the fast-start response of the three closely-related damselfishes, but its effect differed markedly among species. The Ward’s damselfish (*Pomacentrus wardi*) was most affected by water from degraded coral, and displayed shorter distances covered in the fast-start and slower escape speeds compared to fish in water from healthy coral. In the presence of alarm odours, which indicate an imminent threat, the Ambon damsel (*P. amboinensis*) displayed enhanced fast-start performance in water from healthy coral, but not when in water from degraded coral. In contrast, while the white-tailed damsel (*P. chrysurus*) was similarly primed by its alarm odour, the elevation of fast start performance was not altered by water from degraded coral. These species-specific responses to the chemistry of degraded water and alarm odours suggest differences in the way alarm odours interact with the chemical landscape, and differences in the way species balance information about threats, with likely impacts on the survival of affected species in degraded habitats.

## Introduction

Habitat degradation is one of the foremost global causes of extinction^1–3^, but while the loss of organisms due to changes in the quality and characteristics of habitat is well documented^4, 5^, the mechanisms that underlie these changes are often unclear^6^. As a habitat patch degrades the structural complexity of the patch also changes as dominant habitat-forming organisms are lost. These structural changes can affect the utility of visual information, and changes in surface contours can disrupt the flow of air or water moving through a habitat patch, thereby altering the provision of olfactory or sound information^7, 8^, the cues that animals use to inform their behavioural choices^9, 10^.

The partial loss of one community and the colonization by organisms better suited to the new conditions also changes the landscape of chemicals against which the biologically significant odours are interpreted^11^. Alterations to the quality, type or balance of information sources on which to make decisions can lead to making the wrong choice and increase mortality^12–14^. While these changes are becoming increasing commonplace to all habitats, whether terrestrial or aquatic, they currently manifest most dramatically within one of the world’s most biologically diverse and endangered ecosystems, coral reefs^15^.

Coral reefs around the world have been impacted by human induced environmental changes including elevated temperatures, increased frequency of severe storms, modified chemistry and elevated turbidity^16, 17^. These and other changes have seen many coral reefs change from coral dominated to algal dominated landscapes worldwide^5, 18, 19^. Recent studies of coral reef fishes have highlighted how the change from a hard coral to algal dominated landscape can lead to changes in the availability of information on which decisions are made and hence change the balance of information sources used to inform decisions^10, 20, 21^. For organisms that live under a constant threat of predation, how individuals judge risk is central to how they balance vigilance against other fitness promoting behaviours such as foraging, courtship and mating.

Juvenile marine fishes typically employ behavioural tactics to maximize growth^22^, and optimizing when they will respond to a potential predator is critical for maximizing long-term fitness, which is often size-dependent. A fish that waits to the last moment before responding to a predator maximizes its time spent on fitness-promoting activities, but must balance this against a predicted probability of escaping a strike. Until recently it was often assumed that prey escape responses were an all-or-nothing burst of activity that was largely autonomic, with performance maximised by strong predator selection and with little variation within individuals^23–25^. Recent studies have broken up the fast-start escape sequence into parts that are under behavioural modification and those that are more autonomic^26, 27^. For instance, whether to respond and the time to react may be considered as being under some behavioural control, while acceleration and maximum speed are physiologically determined and may be more automatic^26^. For fishes, information to forewarn of the activity of predators in the vicinity often comes from the direct receipt of olfactory, visual or mechanical cues. A recent study found that fish can optimize their fast-start escape performance based on information available concerning the levels of threat posed by a predator^28^. Anything that alters the availability of public information or the perception/detection of the cues, will modify the balance of information used to inform a decision and may alter the ability of a prey to escape a predator strike.

This finding takes on even greater significance when paired with recent evidence showing that chemicals from degraded coral habitats negate the innate response of some fishes to damage-released odours from conspecifics^29^. Fishes of the same, closely-related or ecologically similar species elicit an innate antipredator response to these alarm odours because they represent a reliable indicator of an active predator in the vicinity^30, 31^. Research to date has shown that the ability to use alarm odours in degraded habitats is species specific, with one species no longer able to use alarm odours to inform risk (Ambon damselfish, *Pomacentrus amboinensis*), while another species (the neon damsel, *P. coelestis*) was not adversely affected^29^.

The present study aimed to determine whether a change in the chemical landscape associated with coral degradation affected the way three closely related fish species used olfactory information to optimize their fast-start response when startled. Specifically, we conducted a laboratory experiment that crossed fish species, which have different habitat associations (*P. amboinensis, P. chrysurus, P. wardi*), with background odour (live coral or degraded-dead coral habitats) and threat-forewarning odour (chemical alarm odour or seawater) in a 3 × 2 × 2 design. Fish within each treatment combination were exposed to a mechanized drop stimulus to elicit a startle response. Given our previous finding of a species-specific response to alarm odour in degraded habitats^29^, we predicted that water that had passed over degraded habitat may modify the alarm odour to nullify the forewarning effect in some species, while others may not be affected.

## Methods

### Ethics statement

Research was carried out under approval of the James Cook University animal ethics committee (permit: A2005, A2080) and according to the University’s animal ethics guidelines.

### Study species

Three species of damselfish (Pomacentridae) were used, representing a range of habitat preferences. The Ambon damselfish, *Pomacentrus amboinensis* is a generalist found associated with variety of substrata from live coral through to rubble as a juvenile^32, 33^. The white-tail damselfish, *Pomacentrus chrysurus*, is a dead coral-rubble specialist^34^. The Ward’s damselfish, *Pomacentrus wardi*, is a habitat generalist with a particular affinity for soft coral^35^. The Ambon damselfish is an omnivore feeding on plankton and benthic algae^33^, while the other two species principally feed on benthic algae as adults^34, 35^. All fishes appear to have similar pelagic larval durations^36^.

All fishes were collected as newly metamorphosed juveniles at night using light traps^37^ moored more than 30 m from a reef edge in 10–16 m of water column (trap entrances ~1 m depth) around Lizard Island on the northern Great Barrier Reef (14°40′S, 145°27′E), Australia in October 2015. Fishes were taken from the traps and transported in 60 L tanks to the research station, where they were sorted to species. At testing fish were of similar sizes (standard length): Ambon damsel (range 10.9–14.4 mm, mean 12.5 ± 1.5 mm SE); white-tailed damsel (range 12.1–15.3 mm, mean 13.9 ± 1.2 mm); Ward’s damsel (range 11.9–15.1 mm, mean 13.2 ± 1.3 mm).

### Conditioning treatment

**Figure 1:**
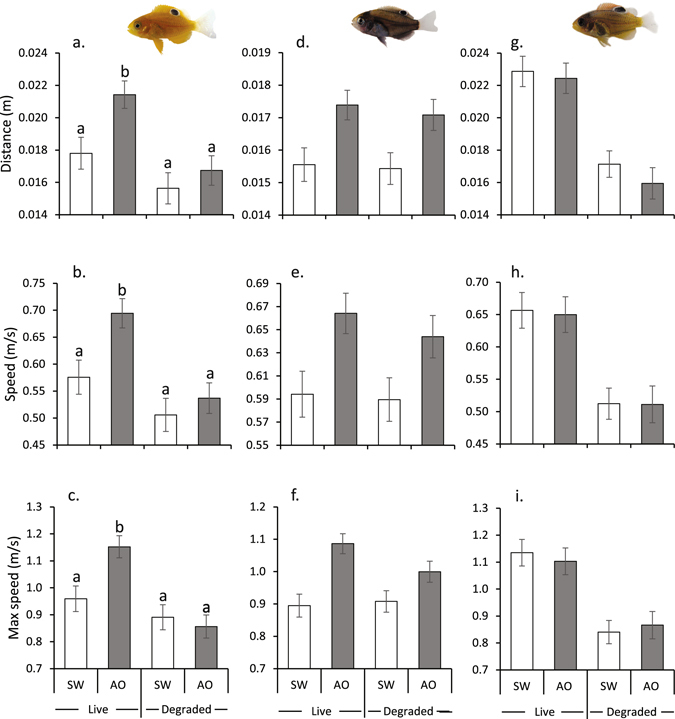
Comparison of the mean (±SE) fast-start performance of three damselfish species, the Ambon damsel (*Pomacentrus amboinensis*; **a**–**c**), the whitetail damselfish (*P. chrysurus*; **d**–**f**) and the Ward’s damselfish (*P. wardi*; **g**–**i**) that had been kept in either water that had passed over live coral or degraded-dead coral, and then exposed to either clean seawater (SW) or conspecific alarm odours (AO) prior to a repeatable startle stimulus. Fast-start variables shown are: response distance (**a**,**d**,**g**), response speed (**b**,**e**,**h**) and maximum speed (**c**,**f**,**i**). N = 17–23. Letters above the bars in panels a–c represent Tukey’s means comparison groupings (only tests on significant interactions between factors are shown, p < 0.05).

Prior to fast-start trials commencing, fishes were randomly allocated to 8 tanks (4 tanks per seawater source) supplied by one of two aerated seawater sources for 48 to 72 h: seawater that had flowed through 35 L header tanks filled with an ~50 cm perimeter piece of live coral (*Pocillopora damicornis*), or seawater that had flowed over dead-degraded coral (dead hard coral and rubble with a similar topography to the live coral but which was covered in algae and some sessile invertebrates; see Fig. 1 McCormick & Lönnstedt^29^ for habitat image). Flow rates were ~1 L per min. Fishes were fed *Artemia* nauplii *ad libitum* and had access to ample shelter during the habitat conditioning period.

### Fast-start arena and protocol

Following conditioning, fishes were transferred individually into a fast-start arena containing water from the appropriate source (i.e., seawater that had passed over live coral or degraded-dead coral) and allowed to acclimate for 5 min. A chemical odour was then introduced into the fast-start arena, and fish were given another 5 min prior to release of the startle stimulus. Fish were only tested once and were tested in water in accordance with the water source they had been conditioned within.

The testing arena consisted of a transparent circular acrylic arena (diameter 200 mm; height 70 mm), within a large opaque-sided plastic tank (585 × 420 × 330 mm; 60 L) with a transparent Perspex bottom to allow responses to be filmed from below as a silhouette (Fig. S1). The water level was low (60 mm) to reduce movements in the vertical plane. Fishes were not fed overnight (~16 h) and after the acclimation period, one of two treatments were applied to the arena: damage-released odours (alarm odours) from conspecifics, or a seawater control (injection control). Fifteen mL of either odour were slowly injected into the inner arena through a piece of clear tubing.

The fish were exposed to either odour for a period of 5 min before a fast-start response was elicited by the release of a weight with a tapered end into the testing arena. Fish were only startled when they moved to the middle portion of the tank, allowing an individual to move an equal distance in any direction and standardising for fish position relative to the stimulus. The weight was released from an electromagnet and was governed by a piece of fishing line that was long enough such that the tapered tip only just touched the surface of the water. To avoid a premature escape response associated with visual stimulation occurring, the weight was released from above into a 550 mm piece of 40 mm diameter PVC pipe with the bottom edge at a distance of 10 mm above the water level. The arena was filled with water from the appropriate source for the trial (i.e., seawater that had passed over live coral or degraded coral) and after each trial the arena was emptied and rinsed to avoid a build-up of alarm odours.

Prey escape variables were only measured when prey performed a C-start (commencement of fast-start that results in the individual forming a C-shape). Escape responses were recorded at 480 frames per second (Casio EX-ZR1000) as a silhouette from below obtained through pointing the camera through a small hole in a black plastic sheet, to avoid visual interference to the fish, at a mirror angled at 45° below the arena. A 1 cm line was drawn in the centre of the inner arena to enable calibration during video analysis.

### Kinematic variables

Kinematic variables associated with the fast-start response were analysed using the image-analysis software Image-J, with a manual tracking plug-in. For analysis of the high-speed movements each fish was reduced to a single moving point. The point where each fish was tracked was standardised by following the same point on each fish (i.e., the position directly behind the eyes which corresponds to the widest part of the body). We chose to standardise tracking based on this point of the body as it the most stable and easiest to track owing to the small size of the juveniles. The following kinematic variables were measured:

1. Response latency (s) was measured as the time interval between the stimulus touching the water surface and the first detectable movement of the fish.
2. Response distance (m) is a measure of the total distance covered by the fish during the first two flips of the tail (the first two axial bends, i.e. stages 1 and 2 defined based on Domenici and Blake^38^, which is the period considered crucial for avoiding ambush predator attacks^39^.
3. Response speed (m/s) was measured as the distance covered within a fixed time (24 ms). This fixed duration was based on the average duration (22.8 ms) of stage 1 and 2 (as defined above).
4. Maximum response speed (m/s) was measured as the maximum speed achieved at any time during stage 1 and stage 2.

### Production of alarm odours

Conspecific alarm odours were used fresh and obtained by making six vertical cuts on each ssof four freshly euthanized (using cold shock) conspecific fish and then rinsing the fish in 60 mL of seawater (of the appropriate source). We injected 15 mL of this alarm odour solution into the fast start arena, which gave a standardised concentration of 2 cuts/L once injected. This concentration has been shown to elicit strong antipredator responses in coral reef fishes^40^.

### Statistical analyses

To determine whether water source (Habitat odour: live coral or dead-degraded coral water) or threat odour (Threat odour: SW or Alarm odour) affected the kinematics of the fast-start response two-factor MANOVA’s were undertaken incorporating all four kinematic variables for each of the three fish species. ANOVA’s were then used to determine the nature of significant differences found by MANOVA. When necessary, Tukey’s HSD post-hoc means comparisons were undertaken. Data were examined for the assumptions of homogeneity of variance and normality using residual analysis. Latency was non-normal and was normalized using a log10(x) transformation. Sample sizes for the four treatment combinations were between 17 and 23 independent fish.

## Results

Seawater from the two sources (from live or dead-degraded coral) and the addition of two threat odours (SW or Alarm odour) influenced the way the three damselfish species reacted to a startle stimulus in different ways (Fig. 1, Sup Fig. S2, Table 1). Within each of the species all kinematic variables displayed the same patterns of significance when examined using ANOVA (Fig. 1, Table 2), with the exception of latency to respond, which was not affected by either factor (p > 0.05; Fig. S2).

**Table 1:** Comparison of the fast-start response kinematics of three juvenile damselfishes that have been kept to seawater that has passed over live-healthy coral or dead-degraded coral (Habitat), and then either exposed to either clean seawater or conspecific alarm odours (Cues) within the burst arena just prior to being startled.

Source	*P. amboinensis*		*P. chrysurus*		*P. wardi*	
	F_4,64_	p	F_4,68_	p	F_4,73_	p
Habitat	3.35	**0.015**	0.46	0.768	14.80	**<0.001**
Cue	2.19	0.080	5.52	**<0.001**	1.25	0.300
Habitat*Cue	2.59	**0.045**	1.01	0.411	1.62	0.180

Damselfish belong to the genus *Pomacentrus*. Results shown are the results of MANOVA’s on four kinematic variables (see text). The test statistic is Pillai’s Trace.

**Table 2:** Comparison of the fast-start response kinematics of three juvenile damselfishes that have been kept in seawater that has passed over live-healthy coral or dead-degraded coral (Habitat), and then either exposed to either clean seawater or conspecific alarm odours (Cues) within the burst arena just prior to being startled.

Species (df)	Variable	Habitat		Cue		Habitat*Cue	
		F	p	F	p	F	p
*P. amboinensis*	Distance	13.6	**<0.001**	4.5	**0.01**	1.8	0.18
1, 75	Speed	14.8	**<0.001**	6.4	**0.01**	2.2	0.14
	Max speed	16.7	**<0.001**	3.2	0.08	6.5	**0.01**
*P. chrysurus*	Distance	0.2	0.66	12.8	**<0.001**	0.04	0.85
1, 74	Speed	0.4	0.51	11.1	**0.001**	0.2	0.68
	Max speed	1.3	0.27	18.4	**<0.001**	2.3	0.14
*P. wardi*	Distance	44.5	**<0.001**	0.8	0.38	0.2	0.68
1, 77	Speed	27.5	**<0.001**	0.02	0.89	0.01	0.92
	Max speed	30.3	**<0.001**	0.004	0.95	0.4	0.55

Damselfish belong to the genus *Pomacentrus*. Results show the outcome of two-factor ANOVA’s on three kinematic variables: response distance (m), speed (m/s) and maximum speed (m/s). Bold values are significant at 0.05.

When the Ambon damselfish was startled in the presence of an alarm odour it showed a marked increase in fast-start performance (Fig. 1a–c), but only in the presence of the alarm odour in water from live coral (MANOVA interaction: F_4,64_, p = 0.045, Table 1). The significant interactions in the multivariate analyses were supported by the Tukey’s comparisons for response distance, speed and maximum speed (Fig. 1a–c). There was no effect of alarm odour when fish had been living in water that had passed over degraded coral.

The whitetail damselfish was affected by the odour added to the water, with higher performance observed when a alarm odour was added to the arena (MANOVA Cue: F_4,68_ = 5.52, p < 0.001; Fig. 1d–f, Table 1). There was no effect of water source on their fast-start response, with a similar magnitude of increase in performance regardless of water source with the addition of the alarm odour in response distance, speed and maximum speed (Fig. 1d–f; Table 2).

In contrast, the Ward’s damselfish was not affected by which cue was added to the water (Fig. 1g–i), but water source had a marked effect (MANOVA Habitat: F_4,73_ = 14.80, p < 0.001, Table 1). Fish displayed higher performance in water that had been in contact with live coral compared to degraded coral and this was shown by response distance, speed and maximum speed (Fig. 1g–i, Table 2).

## Discussion

While recent studies have shown that water that has passed over dead-degraded coral habitat reduces the efficacy of alarm odours in at least one species of coral reef fish^29, 41^, this is the first study to demonstrate that it alters the performance of the fast-start response, a behavior that is crucial for escape from predators. Water from degraded coral habitats affected the fast-start performance of three closely-related damselfishes, but its effect differed markedly among species. The Ward’s damselfish was most affected by water from degraded coral, and displayed shorter distances covered during the fast-start and slower escape speeds compared to fish in water from healthy coral. When the Ambon damselfish was exposed to chemical alarm odours, escape performance was enhanced in keeping with predictions, but only when in a healthy coral water source. Water that had passed over degraded coral canceled the apparent priming effect of the alarm odour. In comparison, while the white-tailed damselfish was similarly primed by its alarm odour, the elevation of fast start performance was not negated by water from degraded coral. These species-specific responses to the chemistry of degraded water and to chemical alarm odours are surprising and suggest differences in the way alarm odours interact with the chemical landscape, and differences in the way species balance information about threats.

Surprisingly, the fast-start performance of the Ward’s damselfish was reduced by ~25% when exposed to water that had passed over degraded coral, while the seawater source alone had no effect on the other two congenerics. Changes to the fast-start response of less than this magnitude have been observed in response to elevated CO_2_ and temperature and been shown to affect survival (e.g., refs 42 and 43). Why the Ward’s damselfish are so affected by water from degraded coral is unclear. Latency to react was not affected, suggesting that the water did not alter the triggering of the Mauthner neurons that initiate the rapid C-start escape response^23^. Clearly the three species have differential sensitivity to chemicals that are active within the water from degraded corals and at least one component of this chemical cocktail appears to affect the Ward’s damselfish.

The provision of chemical alarm odours in the arena prior to the startle stimulus increased the efficacy of the fast-start response in the Ambon and white-tailed damselfish. This forewarning effect may occur because the chemical alarm odours represent a reliable indicator of a threat nearby leading to a priming of the escape response. In general, exposure to a stressor increases circulating catecholamines, such as norepinephrine and epinephrine (in seconds to minutes; ref. 44) and elevates levels of blood cortisol (in minutes to hours). While catecholamines, such as norepinephrine are clear candidates for this priming action given their rapid mobilization and known role in alertness, arousal, and readiness for action^45^, there is currently no data to support their role in fast-start priming^46, 47^. However, studies of freshwater fishes have shown that being exposed to alarm odours triggers a stress response, leading to a relatively rapid increase in cortisol^46–49^, though this is not always the case^50^. Cortisol stimulates gluconeogenesis for the rapid mobilization of glucose into the blood stream. Elevated cortisol primes the body for a rapid response and also allows the body to recover rapidly from the fast start response, which is anerobically fueled and energetically costly^51^. Barreto *et al*.^52^ found that intraperitoneal cortisol implants into the frillfin goby increased its antipredator response to chemical alarm odours, suggesting that cortisol took on a priming function. Moreover, Lastein *et al*.^53^ found that treatment of crucian carp with corticotrophin-releasing factor antagonist before exposure to conspecific skin extracts suppressed the alarm reaction, supporting the hypothesis that cortisol plays a role in the antipredator response displayed to alarm odours. Since cortisol has been shown to heighten neurological activity and speed decisions on simple or well-rehearsed tasks^54–56^, moderate levels of cortisol should enhance vigilance^52^ and the speed of the fast-start response. What is puzzling is that research has previously shown that the Ward’s damselfish displays a typical antipredator response to alarm odours (e.g., ref. 57), yet this alarm odour does not lead to a forewarning effect on the escape response. Obviously further research is necessary to determine the species-specific nature of threat-forewarning for fast-start performance and the physiology that underlies the response.

While the forewarning effect of chemical alarm odours on the performance of fast-starts was seen in two damselfish species, water that had passed over degraded corals prevented the forewarning effect of alarm odours only in one of the species (the Ambon damselfish). In a previous experiment, Lönnstedt *et al*.^20^ rinsed a lacerated alarm odour donor with water from dead-degraded coral and found that odour no longer elicited an antipredator response in the Ambon damselfish, suggesting that the water somehow modified the active components of the alarm odour in this species. However, at least one other damselfish species (the neon damsel, *P. coelestis*) has alarm cues that are not affected^29^ by water from dead-degraded coral. It is currently unknown whether the alarm odours of the Ward’s damselfish still elicit an antipredator response in waters from degraded coral, but the evidence in the current study suggests that it is likely that the alarm odours from the white-tailed damsel are still active in water from degraded coral.

The mechanism by which perception of the alarm odour is disrupted for the Ambon damselfish is poorly understood. Very little is known of the molecular composition of alarm odours. Recently Mathuru *et al*.^58^ found that while zebrafish (*Danio rerio*) respond to a previously suggested alarm odour candidate H_3_NO^59^ with a low intensity antipredator response, a detailed chemical analysis of the alarm odour also found that the components that caused the greatest antipredator response comprised of a mixture of glycosaminoglycans with different molecular weights. Clearly, if this finding is general, then it suggests that the chemicals associated with alarm odours are complex mixtures of compounds that enable them to be species-specific and carry a large amount of information concerning species identity and other information, such as body size and condition (e.g., ref. 60). This complexity argues against a simple olfactory perception model of a physicochemically driven, hard-wired process with specific receptors for alarm odour molecules as suggested by Wilson and Stevenson^61^. Behavioural studies have shown that some fish species will respond with an antipredator response to alarm odours from closely related species, with the intensity of response correlated to their phylogenetic relatedness^62^. This suggests that there is some commonality to the active parts of the alarm odour molecule, but there is sufficient complexity for species-specific identification. Moreover, recent data shows that the Ambon damselfish will respond to the alarm odours of a congeneric *P. nagasakiensis* in water from dead-degraded coral^63^, suggesting that the olfactory receptor sheet is probably not altered by the chemicals from degraded corals. Clearly, the alarm odours of some species, like the Ambon damsel, are adversely affected by degraded water while others have found ways of avoiding this problem. Determining the active ingredients within the degraded reef chemistry and intraspecific variability in the response of sensitive species will be key to determining the extent to which sensitive species may be able to adjust to the prevalence of degraded-dead coral that may surround remnant patches of live coral.

Alarm odours are central to an important cognitive mechanism for learning threats^31^, which is particularly important for aquatic organisms as they transition between habitats and encounter novel predators^64^. Our study has shown that they are also important in that, at least for some species (e.g., Ambon and white-tail damselfish), they play an important role in forewarning species of nearby threats thereby allowing individuals to optimize their fast-start escape response. Interestingly, simply the passage of water past a degraded-dead coral habitat was sufficient to alter the alarm odour of the Ambon damselfish so it no-longer fulfilled a forewarning function, and it also reduced the efficacy of the fast-start response in the Ward’s damsel. This highlights the important role that the olfactory landscape of the environment plays in mediating behavioural responses that may have fitness consequences, such as effective predator avoidance. Determining how species are affected by alterations in the olfactory landscape as coral reefs change from hard coral to algal dominated environments will be important to understanding the dynamics of change in the fish community and what characteristics promote resilience of a species to this dramatic change.

### Data availability

Data is available from: doi:10.4225/28/589a9cbd422bb.

## Electronic supplementary material

Figures showing fast-start apparatus and results of effect of treatments on latency for three damselfishes
