# Supplementary material for: Interspecific differences in how habitat degradation affects escape response
Source: Sci Rep. 2017 Mar 27;7:426. doi: 10.1038/s41598-017-00521-0 (PMC5428724; doi:10.1038/s41598-017-00521-0)
Supplement: Supplementary file 1 — Figures showing fast-start apparatus and results of effect of treatments on latency for three damselfishes [file 41598_2017_521_MOESM1_ESM.pdf]

Supplementary information

Interspecific differences in how habitat degradation affects escape response

Mark I. McCormick, Bridie J. M. Allan

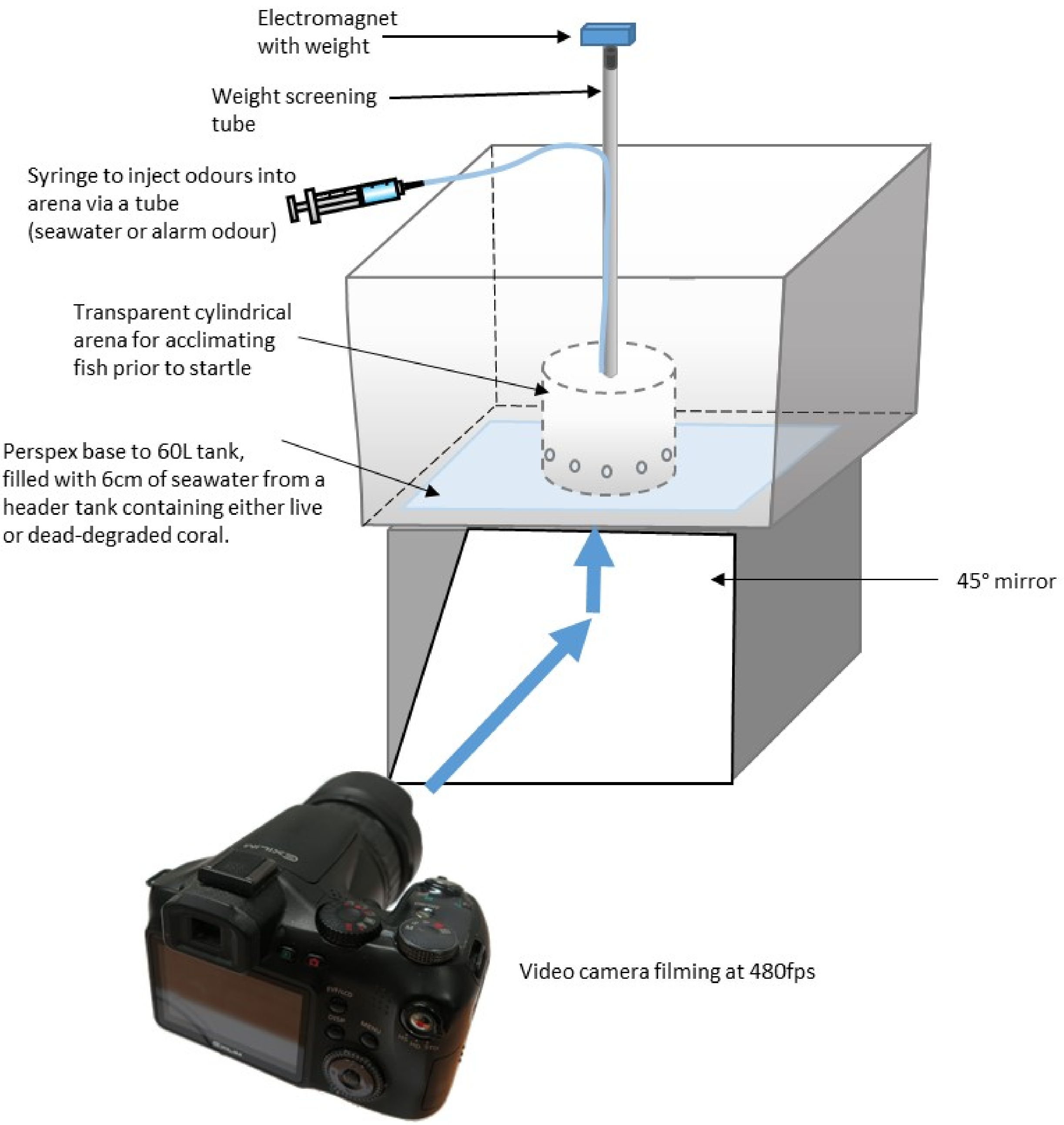

Figure S1. Fast-start arena set-up. One of three species of juvenile damselfish were acclimated for 5 min in the cylindrical arena containing seawater that had passed live or dead coral, then exposed to either an seawater (control) or alarm odour for 5 min, prior to being exposed to a drop startle-stimulus. Camera photograph and diagram elements credit M. McCormick.

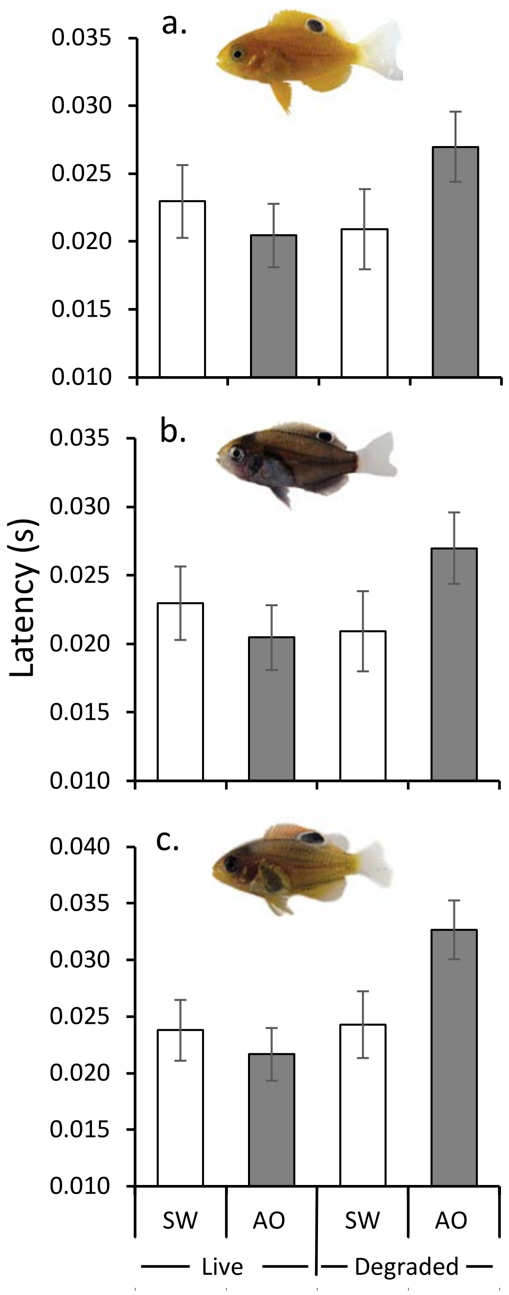

Figure S2. Mean latency (± SE) of the fast start response to a startle stimulus of 3 species of juvenile damselfish: a) Ambon damsel *Pomacentrus amboinensis*, b) white-tail damsel *P. chrysurus*, c) Ward's damsel *P. wardi*. Fish were acclimated to water that had passed over live or dead-degraded coral for 2 days, then exposed to conspecific alarm odours (AO) or a seawater control (SW) just prior to the startle stimulus. N = 17-23. Photographs by M. McCormick.
